# A qualitative thematic content analysis of medical students’ essays on professionalism

**DOI:** 10.1186/s12909-017-0920-5

**Published:** 2017-05-03

**Authors:** So-Youn Park, Changwoo Shon, Oh Young Kwon, Tai Young Yoon, Ivo Kwon

**Affiliations:** 1 Department of Medical Education and Humanities, School of Medicine, Kyung Hee University, 26 Kyungheedae-ro, Dongdaemun-gu, Seoul, Republic of Korea; 2 Department of Urban Society Research, The Seoul Institute, 57, Nambusunhwan-ro 340-gil, Seocho-gu, Seoul, Republic of Korea; 3 Department of Medical Education, School of Medicine, Ewha Womans University, 1071 Anyangcheon-ro, Yangcheon-gu, Seoul, Republic of Korea

**Keywords:** Professionalism, Medical students, Medical education, Korea, Cultural differences, Curriculum development

## Abstract

**Background:**

Physicians in both Western and Eastern countries are being confronted by changes in health care delivery systems and medical professionalism values. The traditional concept of “In-Sul” (benevolent art) and the modern history of South Korea have led to cultural differences between South Korea and other countries in conceptualizing medical professionalism; thus, we studied medical students’ perceptions of professionalism as described in essays written on this topic.

**Methods:**

In 2014, we asked 109 first-year medical students who were enrolled in a compulsory ethics course to anonymously write a description of an instance of medical professionalism that they had witnessed, as well as reflecting on their own professional context. We then processed 105 valid essays using thematic content analysis with computer-assisted qualitative data analysis software.

**Results:**

Thematic analysis of the students’ essays revealed two core aspects of professionalism in South Korea, one focused on respect for patients and the other on physicians’ accountability. The most common theme regarding physician–patient relationships was trust. By contrast, distributive justice was thought to be a non-essential aspect of professionalism.

**Conclusions:**

In Western countries, physicians tend to promote justice in the health care system, including fair distribution of medical resources; however, we found that medical students in South Korea were more inclined to emphasize doctors’ relationships with patients. Medical educators should develop curricular interventions regarding medical professionalism to meet the legitimate needs of patients in their own culture. Because professionalism is a dynamic construct of culture, medical educators should reaffirm cultural context-specific definitions of professionalism for development of associated curricula.

## Background

Although physicians need to learn about medical professionalism [1, 2], there is no scholarly consensus on how to define this concept in South Korea. Superficial education on the topic that is not sufficiently grounded in either core ethical ideas or moral values can cause medical students to develop negative perceptions of professionalism [3]; thus, related medical curricula can end up comprising only lists of rules and/or prohibited behaviors [4].

There are also cultural aspects that must be taken into account when defining medical professionalism because despite considerable advances in medical technology, physicians worldwide are experiencing difficulties in adjusting to changes in health care delivery systems and maintaining traditional professionalism values while also acknowledging patients’ legitimate rights. In Western countries, professionalism was developed on the basis of medicine’s contract with society [5]. However, except for oriental medicine, the medical history of South Korea is relatively short, and the traditional concept of “In-Sul” (benevolent art) which emphasizes the character of a doctor has been the basis of professionalism in this country [6]. Furthermore, physicians in South Korea were not allowed sufficient time to develop their own concept of professionalism after the Korean War. In the 1960s, South Korea had to handle the aftermath of the war. At least 70% of medical facilities were destroyed during the devastating war, and setting the health care system straight was one of the most important tasks for the government [7]. Because of the budget deficit and shortage of doctors, illegal medical practice by unlicensed practitioners was rampant in the country. The government tried to control it by enacting the first medical law – the National Medical Services Law - in 1951 [7]. However, the law was laid more stress on the government control for mobilization of the doctors instead of inducing medical professionalism. Consequently, physicians in South Korea accepted concept of professionalism used in Western countries without modification.

In this paper, we analyzed essays that South Korean medical students wrote about an instance of professionalism that they had witnessed, as well as their reflections on their own professional context. The aim of this study was to take the first step in refining the concept of professionalism in South Korea by examining the distinctive moral convictions that exist within this cultural context.

## Methods

In 2014, we asked 109 first-year medical students, who were participating in a medical ethics course at Kyung Hee University, to complete the following assignment: “In two pages, describe an episode of professionalism you have experienced.” Students were asked to address what had happened and then reflect on their own professional context. They also were directed to consider the consequences of the episode, that is, the most important core ideas for medical professionalism, specific to the described context. We collected and analyzed 105 valid responses (49 females and 56 males, age ranged from 23 to 31, mean age = 25.7) with 96% response rate.

We used computer-assisted qualitative data analysis software to manage and inductively code the essays. QSR NVivo version 10.0 (QSR International Pty Ltd., Doncaster, VIC, Australia) was used to obtain frequency counts and the essays were thematically analyzed. We began with categories based on a conceptual framework developed from the Medical Professional Project by the European Federation of Internal Medicine Foundation, the American College of Physicians and American Society of Internal Medicine, and the American Board of Internal Medicine [5]. Codes under the first four categories in Table 1 were developed through a previous review of the literature [5, 8–10].

**Table 1:** Thematic Content of Medical Students’ Professionalism Essays

Theme	Subcategories
Primacy of patient welfare	Altruism
Physician–patient relationships	Confidentiality
	Respect for patients’ autonomy
	Truth telling/Honesty
	Trust
	Communication
Social justice	Distributive justice
Other physicians’ responsibility	Professional competence
	Professionals’ autonomy
	Work collaboratively with other professionals

The reliability of codes within each category was determined by two phases. First, two coders who were trained in qualitative data analysis methods coded 22 (20%) randomly selected essays. Inter-coder reliability was measured through the kappa statistic [11] and disagreements between the two coders were discussed and solved by consensus. After the codes had been revised, the kappa value was 0.67. Then, the primary coder (CS) processed all of the data using the revised coding scheme.

## Results

**Fig. 1:**
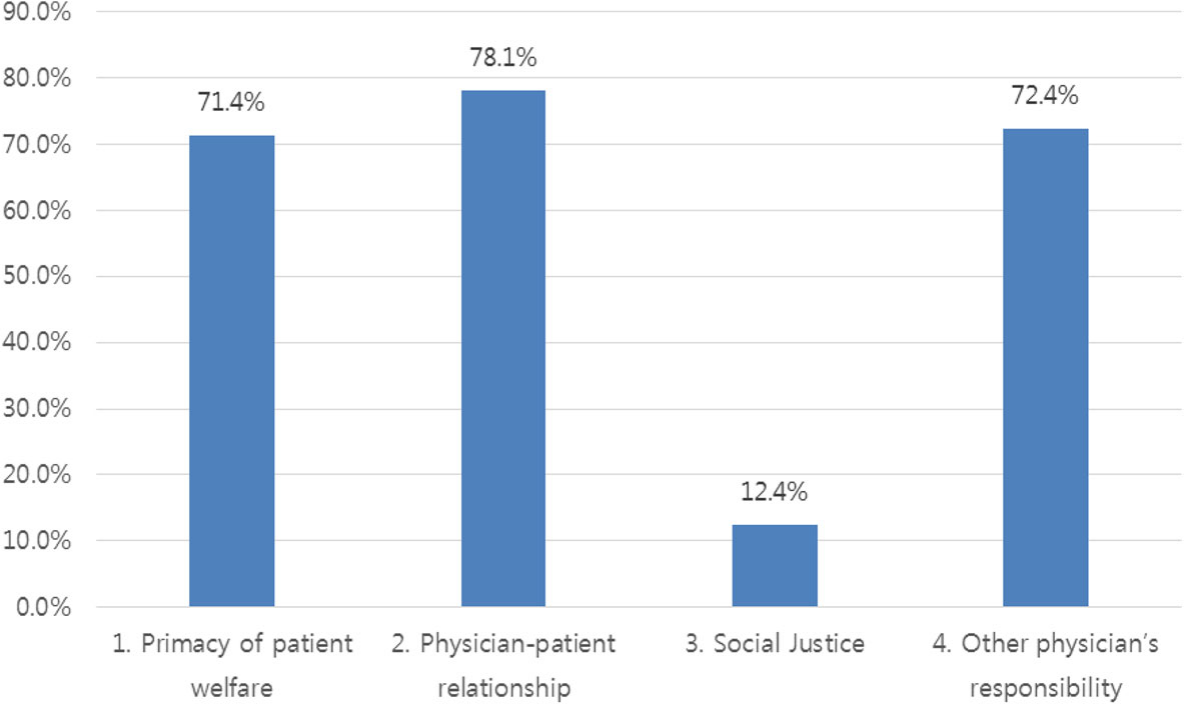
Outline of coding scheme

Figure 1 presents response frequencies with regard to the four identified themes within the concept of professionalism: (1) primacy of patient welfare, (2) physician–patient relationships, (3) social justice, and (4) other physicians’ responsibility.

### Primacy of patient welfare

Overall, 71% (75/105) of the essays described the need for a doctor to think about the best interest of patients in the health care system. One essay described a doctor who routinely prescribed intravenous fluid. The student (#103) wrote the following:

“His attitude of forcing unnecessary medicine was seen as an attempt to pursue his own interests. His clinic is located nearby our university and he has received much criticism in social media for not being dedicated to serving the interests of the patient. […] Physicians must take account of the best interests of a patient above everything else.”

Most students who mentioned altruism emphasized that a doctor should try to understand and put himself or herself in the patient’s shoes.

### Physician–patient relationships

In the essays, 78% of students (82/105) described the physician–patient relationship as one of the important themes of professionalism. This theme comprises the following elements: patients’ confidentiality (4.9% [4/82]), physician–patient communication (51.2% [42/82]), respect for patients’ autonomy (24.4% [20/82]), trust (56.1% [46/82]), and honesty (37.8% [31/82]). Among these, trust was recognized as the most important in relation to the physician–patient relationship. One student (#17) wrote the following:

“Physicians in South Korea have lost the moral authority over patients and, instead of cooperating, they compete with their colleagues. I think that the most urgent thing in this time of crisis is behaving in ways that gain public trust and confidence. […] After that, physicians can state their demands for society based on popular support.”

The other frequent essay theme regarding the physician–patient relationship was communication. One student (#81) who had undergone laser-assisted sub-epithelial keratectomy surgery, reported receiving no help with his or her fear:

“I heard it is a very simple surgical procedure that will take only 5 minutes; however, the pain was more severe than I had expected. I thought the surgery would never finish […] I was in fear during the surgery […] If the doctor had just asked ‘Do you feel all right?’ I would have been more comfortable with the treatment.”

The student then described her idea of a good doctor:

“I experienced that saying something to empathize with a patient’s feelings is one way to achieve professionalism […] I want to be a doctor who shares the patient’s feelings and communicates in the language of the patients.”

Students also reported believing that physicians should ensure that patients are honestly informed before and after treatment. One student (#59) described an episode in which a member of family did not receive a cure because of a medical error. Even though the student conceded that sometimes doctors can make a mistake, she (or he) commented that this doctor did not accept personal responsibility for his error.

“[In this case] the misdiagnosis itself might have been an ‘accident’; however, after the error had been disclosed to our family, the doctor didn’t accept responsibility for it. […] This is an intentional evasion of his responsibility. I think it is an obvious violation of professional ethics.”

Other students wrote that physicians should have respect for patients’ autonomy and fulfill their commitment to confidentiality. Descriptions of manifesting respect in clinical interactions with patients included explaining the need for a certain test or treatment so that the patient could decide about his or her own care. Most of the essays concerning confidentiality included avoiding disclosure of patients’ information. For example, one student (#42) described his experience when he served in the army:

“At that time, I had several symptoms because of hemorrhoids. […] In a public place, one of the military doctors mentioned the details of my disease under the pretense of having given a wrong prescription. Because an army is a very hierarchical society he might not feel guilty about it, but I felt a deep sense of shame. […] Now I spend long days working in a hospital and I’ve sometimes been surprised by the disclosure of patients’ information through gossip by physicians or other health care professionals.”

### Social justice

Overall, 12% (13/105) of the essays described the need for a doctor to promote justice—especially distributive justice—in the health care system. In general, physicians are regarded as having a duty to protect the population’s health and to promote justice in health care contexts [5, 12]. Many physicians in South Korea, however, attach more value to promoting a patient’s health through a form of paternalism, which is also found in the educational goals of medical schools in this country [13]. According to Kim, medical schools seek to establish the Korean doctor’s role in professionalism across four categories: patient care based on ethics and autonomy; doctor–patient relationships; professionalism and self-management; and self-regulation led by professionals. However, distributive justice is not included in these categories. Similarly, in this study, medical students were relatively more inclined to emphasize other categories of professionalism than justice.

### Other responsibilities of physicians

In the essays, 72% (76/105) of the students described the physicians’ responsibility as one of the significant themes of medical professionalism. This theme comprises the following elements: professional competence (81.6% [62/76]), professionals’ autonomy (36.8% [28/76]), and work collaboratively with other professionals (22.4% [17/76]). With regard to professional competence, medical students in this study reported feeling that it is supremely important to uphold scientific standards as well as standards of appropriate use. They noted that physicians should be committed to lifelong learning and dedicated to maintaining their medical knowledge and skills.

Students described the need for a physician to uphold professional standards and norms, and be accountable for professional behavior. Physicians were also expected to take part in the processes of self-regulation. One student (#62) noted a newspaper article reporting on whistle-blowing in the Korean Association of Plastic Surgeons, which held a press conference to apologize for some of their physicians’ unprofessional conduct:

“After anesthesia was administered, instead of the famous physician who had consulted with the patient, another physician performed the plastic surgery. This case shows the current crisis of professionalism. […] Fortunately, the association promised to redouble its efforts to promote self-regulation.”

To maximize patients’ care, many students believed that physicians must reaffirm their autonomy, based on self-regulation and self-education.

Working collaboratively with other health care providers to improve the quality of health care was mentioned in 22.4% of the essays. One student (#25), who had visited an orthopedic physician to have a palpable mass on his or her left thigh examined, described an argument between the physician and the nurse:

“After the test, the physician said that aspiration and some medication might improve the pain. […] When he began the intervention, [suddenly] he started screaming at the nurse because there were no prepared alcohol-soaked cotton balls. The nurse had an argument with the doctor. There was no need for the argument, in my view, because they could make more alcohol-soaked cotton balls. However, I was left on the bed with my pants down in cold weather as the argument continued.”

The student believed that disharmony among physicians and other health care providers might be caused by some physicians having bad attitudes toward and disrespect for other professionals. This student suggested that physicians need to change their attitudes to be a good inter-professional clinical team member.

## Discussion

Because physicians’ contract with society is based on medical professionalism, this concept is a critical learning goal in medical education [1, 2]. This appears to be the first study to exclusively explore the ideas that medical students believe are crucial to medical professionalism, in the specific of South Korea. Students reported observing that some physicians treated their patients without respect for their autonomy, exhibited a lack of communication, and disrespected other specialties. To reduce unprofessional behaviors, the students suggested promoting the professional virtues of altruism, trust, communication, and professional competence.

An introspective examination of professionalism from our own perspective is necessary before implementing the associated education in South Korea. Previous studies reported that the Western framework of medical professionalism might not be appropriate for non-western countries [14–17]. For example, in the Arabian context, professional autonomy was important component of medical professionalism [14]. Virtues of Bushido which means “the way of the warrior” were applicable for medical professionalism in Japanese culture [15]. Ho et al. also suggest “a process to build a professionalism framework” that integrates sociocultural contexts [16]. In this study, many medical students believed that trust in an individual doctor is driven by the communication between patient and physician. According to Brody, “as a trust-generating promise (representing commitment to patients’ interests),” professionalism is one of the key precepts that is needed for robust presentation [3]. Further, patients “provide their medical history and allow their bodies to be examined” because they are sure that “physicians place the interest of patients foremost” [3]. However, our data suggest that if a physician does not maximize the professional interaction through communication, e.g., shared decision making, it is hard to know whether or not this is occurring. The findings of this study highlight the importance of education about communication in real-life situations. Barriers to physician–patient communication could be linked to a lack of insight among physicians that there is a promise between health professionals and the public, as well as to a lack of experience in putting this into practice. Furthermore, factors such as Korean physicians’ preconceptions about patients (e.g., that patients should follow a doctor’s recommendation without experiencing any doubt) and the limited consultation hours that are available in South Korea’s national health insurance system are also related to interpersonal communication difficulties.

What is the best way to teach communication with respect to professionalism in medical schools? First, developing a guide for core content that might be used as the foundation for teaching professionalism is needed for medical schools. The Korea Association of Medical Colleges has published several guides for medical education, regarding the learning outcomes of basic medical education (principle-centered and clinical competency-centered skills), clinical performance, and basic clinical skills [18]. Although these guides help students to reach a certain standard of medical knowledge, educational goals in relation to professionalism have not yet been included. In previous research on the curriculum of 41 South Korean medical schools, it was found that professionalism is taught in various ways in these schools [19]. For example, content analysis revealed that the curriculum for professionalism included diverse subject matter, ranging from the attitudes of medical personnel to medical services marketing [19]. However, there is no consensus on the core concept of what professionalism is and how to instill it in medical students. As noted by Fullan, real change might come through the development of a shared meaning of this concept [20].

Second, after preparing the guide, reforming medical education to link this core idea with a set of behaviors should be facilitated. For example, educating students about communication skills in real clinical situations could be a pedagogic method that effectively promotes an improved connection between physicians and their patients. Medical students should learn how to fully integrate their knowledge and skills when providing patient-centered medical care. Ellman and Fortin argued that medical students are unable to participate in all of the important discussions with families and patients on the wards because of the logistics of educational schedules [21]. Thus, there is a need for a revised curriculum that ensures students learn the crucial aspects of communicating with patients.

Finally, the South Korean students in this study emphasized that physicians should change how they recognize patients’ autonomy. Physicians in South Korea have been experienced rapid changes in the understanding of the patient–physician relationship. Just one decade ago, paternalism prevailed and patient autonomy was not a common concept. Even though there has been a shift in physicians’ awareness of patient autonomy after the introduction of the Healthcare Accreditation in 2010 [22], many of the students in this study mentioned that “physicians must put themselves in the patient’s shoes.” This finding suggests that drawing a bigger picture of educational reform for enhancement of respect for patient autonomy is required in South Korea.

## Conclusion

Medical educators should develop curricular interventions regarding medical professionalism to meet the legitimate needs of patients in their own culture. Unlike Western countries, physicians in South Korea are more inclined to emphasize physician–patient relationships. Because professionalism is a dynamic construct of culture, medical educators should reaffirm the cultural context-specific definitions of professionalism for development of associated curricula.

## References

[1] Harden RM (2000). Evolution or revolution and the future of medical education: replacing the oak tree. Med Teach.

[2] Harden JR, Crosby MH, Davis M, Friedman RM (1999). AMEE guide no. 14: outcome-based education: part 5-from competency to meta-competency: a model for the specification of learning outcomes. Med Teach.

[3] Brody H, Doukas D (2014). Professionalism: a framework to guide medical education. Med Educ.

[4] Coulehan J (2005). Viewpoint: today's professionalism: engaging the mind but not the heart. Acad Med.

[5] Medical Professionalism Project (2002). Medical professionalism in the new millennium: a physicians' charter. Lancet.

[6] Kwon I (2012). Medical ethics as professional ethics. Korean J Gastroenterol.

[7] Lee J (2010). State control of medicine through legislation and revision of the medical law: licensed and unlicensed medical practices in the 1950s - 60s. Uisahak.

[8] Hafferty FW, Castellani B (2010). The increasing complexities of professionalism. Acad Med.

[9] Monrouxe LV, Rees CE, Hu W (2011). Differences in medical students' explicit discourses of professionalism: acting, representing, becoming. Med Educ.

[10] Jin P (2015). The physician charter on medical professionalism from the Chinese perspective: a comparative analysis. J Med Ethics.

[11] Landis JR, Koch GG (1977). The measurement of observer agreement for categorical data. Biometrics.

[12] Kniess J (2015). Obesity, paternalism and fairness. J Med Ethics.

[13] Kim DH, Kim EJ, Hwang J, Shin JS, Lee S (2015). What is the current orientation of undergraduate medical education in Korea?. Korean J Med Educ.

[14] Al-Eraky MM, Chandratilake M (2012). How medical professionalism is conceptualised in Arabian context: a validation study. Med Teach.

[15] Nishigori H, Harrison R, Busari J, Dornan T (2014). Bushido and medical professionalism in Japan. Acad Med.

[16] Ho MJ, Yu KH, Hirsh D, Huang TS, Yang PC (2011). Does one size fit all? Building a framework for medical professionalism. Acad Med.

[17] Pan H, Norris JL, Liang YS, Li JN, Ho MJ (2013). Building a professionalism framework for healthcare providers in China: a nominal group technique study. Med Teach.

[18] The Korean Association of Medical Colleges. Guidelines for medical education; Available from: http://www.kamc.kr/main/index.php?m_cd=30. Accessed 2 May 2017.

[19] An JH, Kwon I, Lee SN, Han JJ, Jeong JE (2008). Study on the medical humanities and social sciences curriculum in Korean medical school: current teaching status and learning subjects. Korean J Med Educ.

[20] Fullan M (2007). The new meaning of educational change.

[21] Ellman MS, Fortin AH (2012). Benefits of teaching medical students how to communicate with patients having serious illness: comparison of two approaches to experiential, skill-based, and self-reflective learning. Yale J Biol Med.

[22] The Korea Institute for Healthcare Accreditation (KOIHA). Foundation purpose and History; Available from: http://www.koiha.kr/member/en/contents/ensub01/ensub01_04.do. Accessed 2 May 2017.

